# Artificial Intelligence’s Transformative Role in Illuminating Brain Function in Long COVID Patients Using PET/FDG

**DOI:** 10.3390/brainsci14010073

**Published:** 2024-01-10

**Authors:** Thorsten Rudroff

**Affiliations:** 1 Department of Health and Human Physiology, University of Iowa, Iowa City, IA 52242, USA; thorsten-rudroff@uiowa.edu; Tel.: +1-(319)-467-0363; Fax: +1-(319)-355-6669; 2 Department of Neurology, University of Iowa Hospitals and Clinics, Iowa City, IA 52242, USA

**Keywords:** AI, Long COVID, neuroimaging, cognition, non-invasive brain stimulation

## Abstract

Cutting-edge brain imaging techniques, particularly positron emission tomography with Fluorodeoxyglucose (PET/FDG), are being used in conjunction with Artificial Intelligence (AI) to shed light on the neurological symptoms associated with Long COVID. AI, particularly deep learning algorithms such as convolutional neural networks (CNN) and generative adversarial networks (GAN), plays a transformative role in analyzing PET scans, identifying subtle metabolic changes, and offering a more comprehensive understanding of Long COVID’s impact on the brain. It aids in early detection of abnormal brain metabolism patterns, enabling personalized treatment plans. Moreover, AI assists in predicting the progression of neurological symptoms, refining patient care, and accelerating Long COVID research. It can uncover new insights, identify biomarkers, and streamline drug discovery. Additionally, the application of AI extends to non-invasive brain stimulation techniques, such as transcranial direct current stimulation (tDCS), which have shown promise in alleviating Long COVID symptoms. AI can optimize treatment protocols by analyzing neuroimaging data, predicting individual responses, and automating adjustments in real time. While the potential benefits are vast, ethical considerations and data privacy must be rigorously addressed. The synergy of AI and PET scans in Long COVID research offers hope in understanding and mitigating the complexities of this condition.

## 1. Introduction

The COVID-19 pandemic has fundamentally changed the way we view healthcare, leaving us with a plethora of unanswered questions and emerging challenges. Long COVID, also known as post-acute sequelae of COVID-19, refers to the persistent symptoms experienced by some patients after recovering from an initial COVID-19 infection. There has been much discussion around the terminology used to describe the extended health effects of COVID-19 infection. Terms such as “Long COVID”, “COVID long-haulers”, “post-acute COVID-19”, and “late sequelae of COVID-19” have all been proposed. However, for consistency in this perspective, we will use the term “Long COVID”. The WHO defines Long COVID as a condition occurring in those with a confirmed or probable history of SARS-CoV-2 infection, typically 3 months from initial COVID-19 onset [1]. While the respiratory and cardiovascular aspects of COVID-19 have been widely studied, less attention has been paid to its effects on the brain. Recently, researchers have begun employing cutting-edge imaging techniques, such as positron emission tomography with fluorodeoxyglucose (PET/FDG), in conjunction with artificial intelligence (AI), to delve into the intricate world of brain function in Long COVID patients. This innovative approach has the potential to shed light on the neurological symptoms of Long COVID and may pave the way for more effective treatments.

## 2. The Enigma of Long COVID and Brain Function

There is increasing concern about the potential effects of Long COVID on brain function and cognition. Many Long COVID patients report neurological symptoms including fatigue, headache, loss of taste and smell, impaired concentration and mental fog, forgetfulness, anxiety, and depression. Studies have found objective cognitive deficits in some Long COVID patients, including impaired performance on tests of processing speed, executive function, verbal learning, and episodic memory [2,3,4,5,6]. Neuropsychiatric disorders like anxiety, depression [7], and post-traumatic stress disorder (PTSD) [8] also appear to be more common following COVID-19. The biological mechanisms underlying these cognitive and neuropsychiatric effects are still under investigation but likely involve neuroinflammation, microvascular changes, and neural network dysregulation.

Several theories have been proposed regarding the pathophysiology of Long COVID neurological effects [9]:

- Direct viral invasion of the brain.
- Neurotoxic effects of inflammatory mediators.
- Autoantibodies against neural antigens.
- Microvascular pathology and blood–brain barrier disruption.
- Mitochondrial dysfunction and cellular bioenergetics issues.
- Neuroplasticity changes due to illness stressors.

The neurological manifestations of Long COVID have puzzled researchers, as the virus primarily affects the respiratory system. In an effort to understand the underlying mechanisms, the focus has shifted towards brain imaging techniques like PET/FDG and the power of AI.

## 3. PET/FDG Imaging: A Window into Brain Function

Emerging brain imaging studies are providing insights into potential neurological changes associated with Long COVID. Positron emission tomography imaging with fluorodeoxyglucose (PET/FDG) is emerging as a promising tool for illuminating brain abnormalities associated with Long COVID. PET/FDG scans involve the injection of a radioactive tracer into the body, which accumulates in areas with high metabolic activity, such as the brain. The PET scanner then detects the gamma rays emitted by the tracer, creating a detailed image of the brain’s metabolic activity. PET/FDG provides a non-invasive way to measure glucose metabolism in the brain.

Details on the PET/FDG imaging techniques and protocols typically used to assess brain function in Long COVID studies [10,11,12]:

- Radiotracer used: 18F-fluorodeoxyglucose (FDG), a glucose analog, is the standard radiotracer used to image glucose metabolism in the brain.
- PET scanner types: These studies generally use whole-body PET/CT scanners or dedicated brain PET scanners with a resolution around 4–6 mm.
- Patient preparation: Patients are asked to fast for 4–6 h before the scan to stabilize metabolic state. Serum glucose levels are checked prior to radiotracer injection.
- FDG Dose: 5–10 mCi of FDG is injected intravenously. Scanning begins 30–60 min post-injection when radiotracer accumulation in brain reaches equilibrium.
- Scan duration: 15–30 min per PET acquisition. Longer scans can improve image statistics and allow for full brain coverage.
- Image reconstruction: Iterative reconstruction algorithms like ordered subset expectation maximization (OSEM) used.
- Image processing: Standardized uptake value (SUV) metrics calculated in regions of interest. AI algorithms applied for advanced analyses.
- Control groups: Age-matched healthy controls are scanned using the same protocol for comparison.

Standardization of imaging protocols is important to obtain reproducible quantitative results across subjects and follow-up scans. The combination of PET with AI and MRI scans provides complimentary information on neural inflammation, network disruption, and atrophy patterns in Long COVID.

Areas of decreased metabolism on PET/FDG imaging have been linked to inflammation and neurodegeneration. PET/FDG allows for measurement of glucose metabolism as an indicator of inflammation and cellular activity [10]. It can identify affected brain regions in Long COVID patients. PET/FDG studies have found reduced metabolic activity and hypometabolism in certain brain areas of Long COVID patients, including the frontal and temporal lobe, limbic system, and brainstem. This suggests inflammation preferentially targeting these regions. The brain hypometabolism patterns are associated with neuropsychiatric disorders and could underline cognitive/neurological symptoms in Long COVID [11,12]. The neuropsychologic test battery comprises the Hopkins Verbal Learning Test-Revised, Brief Visuospatial-Memory Test-Revised, Digit Span forward/revers, Trail Making Test part A/B, Color-Word Interference Test, Symbol-Digit Modalities Test, and a semantic and letter fluency test. However, PET/FDG requires interpretation by specialists in nuclear medicine and neuroimaging to make pattern recognition decisions mostly using qualitative readings. Thus, the challenge lies in interpreting the complex data generated by these scans, and this is where AI comes into play.

## 4. Artificial Intelligence (AI) as the Cognitive Enhancer

In this quest for understanding, the marriage of PET/FDG and AI stands out as a transformative force, offering a beacon of hope in our battle against Long COVID. AI has revolutionized the healthcare industry, and its applications extend to the interpretation of medical images. In the case of PET/FDG scans, AI algorithms have demonstrated remarkable capabilities in detecting subtle changes in brain metabolism that might be challenging for human experts to identify. These algorithms can process vast amounts of data quickly and efficiently, increasing the accuracy and reliability of results.

Matsubara et al. [13] reviewed recent studies applying AI, especially deep learning techniques, for PET image generation. For denoising, convolutional neural networks (CNNs) like U-Net [14,15] and generative adversarial networks (GANs) [16,17] have been applied to recover standard dose/duration PET from low-dose/short scans. CNNs have become the predominant deep learning approach for recovering full PET data from low-dose or abbreviated scans. Xiang et al. [18] pioneered the use of CNNs for this application, training a model to generate standard 12 min brain fluorodeoxyglucose (FDG) PET images from 3 min scans. Their auto-context CNN architecture, comprising three 4-layer CNN blocks with skip connections, achieved results comparable to the previous state-of-the-art method. Subsequently, U-Net, a U-shaped CNN with built-in skip connections, has proven highly effective for full PET data recovery across various tracers and scan types. For example, Chen et al. [19] showed a U-Net trained on multi-contrast MRI could recover full-dose amyloid PET scans from just 1/100 of the radiotracer dose. Recovered images enabled accurate visual assessment of amyloid status. U-Net has also been successfully applied to reconstruct full-dose whole body and cardiac PET/FDG images from abbreviated scans.

### 4.1. CNNs and GANs for PET Image Generation

CNNs and GANs are types of deep neural networks with different architectures and applications.

### 4.2. CNN Architectures for PET Image Generation

CNNs are a specialized type of artificial neural network commonly used for image processing and computer vision tasks. Here is a quick explanation of how CNNs work:

- Convolutional layers—These layers perform convolutions over the input image to extract features. The convolution is performed by sliding filters or kernels over the image and computing dot products between the filter and image patch. Different filters detect different types of features like edges, colors, textures, etc.
- Pooling layers—These layers downsample the image representation to reduce computational load and overfitting. Max pooling takes the maximum within filter regions while average pooling takes the average.
- Fully connected layers—These classic neural network layers connect the extracted features to the output nodes for classification. They help combine the features and make predictions.
- Non-linear activations—Non-linear activation functions like ReLU are applied after each convolution and fully connected layer to introduce non-linearity in the model.

Some key advantages of CNNs are the ability to automatically learn relevant features from training data, invariance to translations, rotations and distortions, and capability to exploit spatial structure. CNNs have revolutionized computer vision and are also very effective for neuroimaging analysis and diagnosis. However, they require large-labeled datasets for training. Overall, CNNs provide a powerful tool for automated feature learning from neuroimages. CNN architectures, especially U-Net, have become the dominant deep learning approach for recovering complete PET data from low-dose or short-duration scans. CNNs can generate full dynamic range, standard duration PET images from truncated scans across brain, whole body, and cardiac imaging.

### 4.3. GANs as an Alternative Approach

GANs have emerged as an alternative deep learning approach for recovering full dose, standard-duration PET images from truncated scans. Wang et al. [20] first applied adversarial training between a generator network to produce 12 min brain PET/FDG scans from 3 min scans, and a discriminator network to classify images as real or generated. Their 3D conditional GAN architecture outperformed 3D U-Net in terms of peak signal-to-noise ratio, normalized mean squared error, and standard uptake value bias. Subsequently, Lu et al. [21] demonstrated GANs could reconstruct whole-body PET/FDG images to standard dose levels from just 10% dose scans. The GAN achieved comparable performance to U-Net in terms of signal-to-noise ratio and standard uptake value biases.

Here is a simplified explanation of how GANs work:

- Generator network—This network generates new synthetic data instances (images, audio, etc.) that are similar to the training data. It starts from random noise and transforms it to match the data distribution.
- Discriminator network—This network tries to distinguish between real training data and the synthetic data created by the generator. It estimates the probability that a sample came from the real training data.
- Adversarial training—The generator and discriminator networks are trained together in an adversarial manner. The generator tries to better fool the discriminator, while the discriminator tries to properly classify real vs. fake data.
- Nash equilibrium—The training reaches equilibrium when the generator produces such realistic data that the discriminator cannot differentiate it from real data. At this point, both models have maximized their objectives.

The key advantages of GANs include the ability to generate novel realistic data, learn meaningful latent representations, and model complex high-dimensional distributions. In neuroimaging, GANs can be used for data augmentation, image synthesis, and modeling brain data distributions. In summary, alongside CNNs, GAN frameworks show promise for reconstructing complete, full-dynamic range PET scans from low-dose or short acquisition protocols across brain and whole-body imaging. Adversarial training provides an alternative deep learning strategy to CNNs for PET image recovery tasks. However, training stability can be an issue with GANs. Overall, they are a powerful generative modeling framework with many applications in medical imaging and healthcare.

So, in summary, CNNs are optimized for discriminative tasks while GANs are optimized for generative modeling and synthetic data generation. CNNs classify data while GANs create new data, but they can complement each other in certain applications.

### 4.4. CNNs and GANs for Image Translation and Synthesis

The application of deep learning (CNN, GAN) in medical imaging extends to the challenging tasks of intra- and inter-modality image translation and image synthesis. Techniques like CNN and GAN have successfully tackled these previously daunting endeavors within the medical imaging domain [22]. An illustrative example of this progress is the use of deep learning to create computed tomography (CT) images from magnetic resonance (MR) images, which has been employed to enhance PET attenuation correction in hybrid PET/MR scanners, as elaborated in the “PET attenuation correction” section.

The utilization of deep learning (CNN, GAN) for image translation and synthesis in medical imaging offers three significant advantages for PET imaging:

1. Supplement Missing Data: In medical imaging, missing data can occur due to various reasons. For example, the acquisition of thin-sliced MR images is often omitted from clinical routines due to lengthy scan durations, even though these thin-sliced MR images are essential for quantitative analysis of brain PET images. Deep learning can be employed to synthesize thin-sliced MR images, thus enabling quantitative analysis of PET images even when MR acquisitions are not available.
2. Reduction in Scans: Deep learning-driven image translation and synthesis allow for the avoidance of acquiring specific target images, leading to a reduction in the total acquisition time. This reduction not only alleviates the burden on patients but also minimizes their exposure to radiation.
3. Data Augmentation: Image translation and synthesis play a vital role in data augmentation, addressing issues related to insufficient training data and data imbalance in machine learning applications. This approach is especially valuable in the computer-aided diagnosis of rare diseases where collecting large amounts of data is a formidable challenge. Deep learning-based data augmentation through image translation and synthesis enhances the performance of machine learning models in such cases.

### 4.5. CNNs and GANs for Diagnosis and Prediction

CNNs (convolutional neural networks) and GANs (generative adversarial networks) are two types of deep learning architectures that show promising applications for helping with diagnosis and prediction in Long COVID patients:

1. CNNs for Diagnostic Pattern Recognition: CNNs can be trained on medical imaging datasets like PET, MRI, or CT scans to recognize unique radiographic signatures associated with post-COVID neurological, cardiovascular, or respiratory damage. This allows for automated diagnosis aid systems to be developed for detecting Long COVID sequalae.

For example, brain PET scans analyzed by a CNN could identify distinct patterns of inflammation or glucose hypometabolism that characterize memory and cognitive dysfunction in long haulers.

2. GANs for Synthetic Data Augmentation: A major barrier in applying deep learning is limited patient data. GANs can generate synthetic PET scans that emulate Long COVID-specific abnormalities like neurological inflammation. This artificially expanded dataset helps train CNN diagnostic models to be more robust and generalizable with less real-world examples.
3. CNNs for Prognostic Risk Stratification: Analyzing temporal sequences of scans from confirmed Long COVID patients, CNN algorithms can discover prognostic imaging biomarkers linked to disease recovery trajectories. Such predictive models can guide treatment personalization and follow-up care.

Overall, CNNs and GANs have exciting utilities in harnessing medical imaging data to assist detection, prognosis, and management of Long COVID afflictions. Larger multi-center studies are needed to assemble diverse training data to implement these AI technologies.

### 4.6. Further Readings on CNNS and GANs

Review articles that provide an overview and survey of CNNs and GANs: “Optimizing Image Captioning using Deep Learning based Object Detection” by Sahu et al. [23] provides a thorough review of CNN architectures like VGGNet, ResNet, Inception, etc., for image captioning. “Generative Adversarial Networks: An Overview” by Creswell et al. [24] reviews the basic framework, theory, types of GANs, training methods, evaluation metrics, and applications. “A review of convolutional neural networks for inverse problems in imaging” [25] focuses on CNN methods for image denoising, super-resolution, inpainting, artifact removal, etc. “Convolutional Neural Networks for Medical Image Analysis: Full Training or Fine Tuning?” [26] discusses CNN training techniques for medical imaging—full training vs. fine tuning. “Recent advances in deep learning for medical image segmentation” [27] surveys deep learning especially CNNs for medical image segmentation tasks.

These review papers provide a broad overview of key CNN and GAN methodologies, architectures, applications, and trends within computer vision and medical imaging. They serve as a good starting point to better understand these widely used deep learning techniques.

In summary, deep learning’s ability to perform image translation and synthesis in the field of medical imaging has opened up new avenues for improving the quality and efficiency of various medical procedures, including PET imaging. These advancements have the potential to enhance patient care, accelerate diagnoses, and facilitate the development of more effective treatments. Limitations include lack of large PET datasets and evaluation metrics for generated images. Future directions include unsupervised learning and transformer models. Deep learning has brought advances in PET image generation and will likely be commonly used in clinical practice for image quality improvement and scan burden reduction.

## 5. The Transformative Role of AI in Long COVID Research

AI possesses a unique ability to analyze intricate patterns and subtle changes in PET/FDG data that might evade human recognition. By identifying regions of the brain with altered metabolic activity, AI can offer a deeper understanding of how Long COVID affects the brain. These findings help pinpoint areas of concern, such as inflammation or reduced blood flow, providing a more precise view of the neurological changes associated with the condition.

Where AI truly shines is in its capacity to discern patterns and correlations in vast datasets. Researchers can use AI to compare the PET scans of Long COVID patients with those of healthy individuals, thus revealing specific brain regions that exhibit abnormal activity. These discoveries hold the promise of identifying novel biomarkers associated with Long COVID, allowing for early diagnosis and the development of targeted treatment strategies.

Furthermore, AI can help predict the progression of neurological symptoms in Long COVID patients. By analyzing PET scan data alongside clinical information and genetics, AI models can provide insights into the likelihood of severe cognitive impairment or mental health issues. This proactive approach to patient care offers an invaluable opportunity to manage Long COVID ‘s long-term effects.

AI-powered computational approaches can integrate diverse datasets from omics to imaging to glean new mechanistic insights into Long COVID pathophysiology. For example, deep learning algorithms applied to high-dimensional molecular data may uncover novel biological pathways underlying lingering symptoms. AI-enabled analysis of medical imaging could identify distinct radiographic phenotypes and signatures of organ dysfunction.

Advanced analytics using natural language processing and machine learning on enormous sets of healthcare data can unravel risk factors and subtypes of Long COVID. AI tools can rapidly mine electronic health records, insurance claims data, digital biomarker wearables, and more to find clinical, demographic, and social determinants that predispose patients to Long COVID or its most severe presentations. These big data analytics can guide prognosis, treatment, and study design.

Another application is the accelerated identification of new therapeutics for Long COVID using AI-based drug discovery and repurposing platforms. By screening libraries of molecules, predicting compound–target interactions, and modeling drug response, AI can fast-track the development of novel treatments to alleviate stubborn Long COVID symptoms. AI can also identify promising repurposing opportunities for existing drugs.

AI promises to transform Long COVID clinical trials through better participant stratification and outcome measurement. It also enables tailored interventions via individualized predictions. In the clinic, AI augmentation can help multidisciplinary Long COVID care teams deliver coordinated, evidence-based services. Chatbots and virtual assistants provide accessible support.

Summary points:

1. Early Detection: AI can help in the early identification of abnormal brain metabolism patterns in Long COVID patients, potentially allowing for timely interventions and personalized treatment plans.
2. Precision Medicine: By analyzing PET/FDG scans alongside other clinical and genetic data, AI can facilitate the development of more precise treatment strategies tailored to each patient’s unique profile.
3. Monitoring Disease Progression: Long COVID can manifest differently in various individuals, and its symptoms may evolve over time. AI can continuously monitor brain function and adapt treatment plans accordingly.
4. Accelerating Research: AI-powered analysis of PET/FDG data from a large number of patients can speed up research into Long COVID, enabling a better understanding of the condition and potential therapies.
5. Uncovering New Insights: AI can identify patterns and correlations that might go unnoticed by human researchers, leading to the discovery of previously unknown factors contributing to Long COVID.

AI is a disruptive technology that can substantially advance every aspect of Long COVID research and care—from elucidating biological mechanisms to validating treatments. By leveraging the power of AI, researchers seek to unravel the mysteries of this confounding condition and substantially improve patient outcomes. The full benefits have yet to be realized; however, the future looks bright at the intersection of AI and Long COVID research.

Nonetheless, as we embrace AI’s transformative role in illuminating brain function with PET scans in Long COVID patients, we must also consider the ethical implications. Safeguarding patient data privacy and ensuring responsible AI usage is paramount. Strict measures and regulations should be in place to protect individuals’ rights and personal information.

## 6. Challenges for Clinical Transformation: Beyond Performance Validation

**Figure 1:**
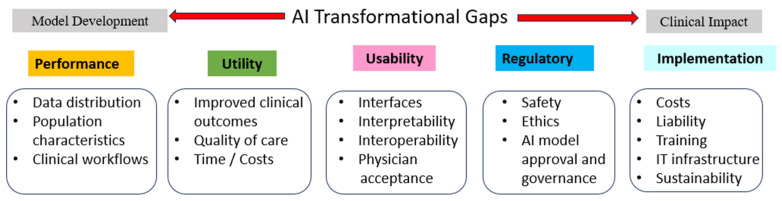
Navigating the AI transformational gap between initial model development and routine clinical Long COVID care by emphasizing and demonstrating five essential concepts: performance, utility, usability, regulatory, and implementation.

There is a significant challenge in translating AI innovations into routine patient care. The transformational gap refers to the gap between developing an artificial intelligence/machine learning model in a research setting and successfully deploying it in real-world clinical practice. Some key aspects of the transformational gap include (Figure 1):

- Performance gap—Models often perform worse in real-world settings compared to research environments due to differences in data distribution, population characteristics, clinical workflows, etc. Bridging this gap requires extensive validation and testing.
- Utility gap—Even accurate models may not improve meaningful clinical outcomes, quality of care, or costs. Clinical utility needs to be proven through randomized trials or comparative effectiveness studies.
- Usability gap—Integration into clinical workflows is non-trivial. Factors like user interfaces, interpretability, interoperability, and physician acceptance determine real-world adoption.
- Regulatory gap—Lack of regulatory frameworks for AI/ML model approval and governance creates uncertainty around safe and ethical deployment.
- Implementation gap—Organizational barriers around costs, liability, reimbursement, training, and IT infrastructure can prevent adoption. Planning for sustainability is crucial.

## 7. Potential Future Research Avenues

### 7.1. Assessing Cognitive Impairment in Long COVID Patients

As discussed above, cognitive impairments like brain fog, difficulty concentrating, and memory issues have emerged as common Long COVID symptoms [2,3,4,5,6,7,8,9]. Assessing cognitive impairment in Long COVID patients is crucial for prognosis and guiding treatment. FDG/PET imaging provides a quantitative means to measure brain metabolism and has shown utility in evaluating neurodegenerative disorders like Alzheimer’s disease [28]. AI techniques like deep learning algorithms offer new opportunities to analyze FDG/PET data to predict cognitive decline.

### 7.2. Using PET/FDG and AI for Early Prediction

PET/FDG brain scans analyzed by convolutional neural networks (CNNs) can be used to predict future cognitive impairment in Long COVID patients. CNNs can extract spatial features from PET images relevant to brain metabolism patterns linked to cognitive decline. By training CNN models on labeled FDG/PET data from cognitively normal and impaired populations, the networks can learn to classify scans based on disease-related metabolic patterns. Long COVID patients, especially those over age 50, complaining of brain fog [28] would undergo FDG/PET scans at baseline. A pretrained CNN classifier would analyze the PET data to generate predictions on the patient’s risk of developing mild cognitive impairment or dementia within 1–2 years. High-risk patients could then potentially be selected for early interventions or clinical trials for Alzheimer’s prevention. The AI could also help uncover why COVID-19 might raise dementia risk in some patients.

Key challenges include curating multi-institutional labeled PET datasets for model training and validation. Physician assessments of cognitive function using standard tests like the Montreal Cognitive Assessment [29] would provide ground truth labels. Extensive testing is essential to establish the predictive performance and clinical utility of the AI methodology.

This AI-powered FDG/PET approach could enable early identification of Long COVID patients at risk for cognitive decline. Early interventions could then be explored to halt further deterioration. With Long COVID affecting millions globally, tools to assess long-term neurological impacts are urgently needed.

### 7.3. Enhancing Non-Invasive Brain Stimulation with AI

Furthermore, non-invasive brain stimulation (NIBS) techniques like transcranial.

Direct-Current Stimulation (tDCS), transcranial Alternating Current Stimulation (tACS), and transcutaneous Vagus Nerve Stimulation (tVNS) can modulate brain activity and connectivity. They are safe, well-tolerated options for neurological disorders. tDCS studies show reduced fatigue and improved cognition in Long COVID patients when applied to frontal and parietal areas [30]. tACS may counteract abnormal brain oscillations underlying fatigue [31]. Early data show cognitive improvements in Alzheimer’s patients. tVNS activated cholinergic anti-inflammatory pathways and reduced fatigue in a Long COVID pilot study [32]. It has anti-inflammatory effects relevant to post-viral immune dysfunction. NIBS provides a promising non-pharmacological approach to target proposed mechanisms underlying Long COVID fatigue like inflammation, hypofrontality, and network dysfunction. More research is needed on optimal NIBS protocols and sham-controlled trials in Long COVID patients, but early findings suggest these techniques could alleviate persistent neurological symptoms.

AI could be utilized to advance NIBS techniques. Machine learning algorithms can analyze neuroimaging scans (PET/FDG, fMRI, EEG) before stimulation to identify optimal target regions for each patient based on their unique brain connectivity patterns.

- AI models can be trained on large datasets to predict individual treatment response and side effects based on demographic, clinical, and neuroimaging variables. This allows for personalized, precision medicine approaches.
- Closed-loop systems can track physiological signals during stimulation and automatically adjust stimulation parameters in real time to optimize effects.
- Reinforcement learning algorithms can iteratively adjust stimulation settings across sessions to maximize therapeutic benefits and minimize side effects for each patient.
- Advanced neural networks and deep learning models can help automate analysis of complex physiological signals acquired during and after stimulation.
- AI planning can design optimal stimulation protocols involving scheduling, electrode placement, and dosage to efficiently achieve treatment goals.
- Big data analytics can identify patterns, correlations, and subgroups across diverse patient populations that inform individualized stimulation protocols.
- Simulations of brain network dynamics can model effects of stimulation on connectivity. This allows for in silico optimization before delivering it to patients.
- Natural language processing can extract clinically meaningful insights from patient reports on symptoms over the course of therapy.

In summary, AI has diverse applications spanning predictive modeling, closed-loop control systems, large-scale analytics, simulations, and adaptive learning algorithms that can enhance development of non-invasive brain stimulation as a precision medicine for neurological disorders.

## 8. Limitations

Evaluating the quantitative accuracy of PET images generated by deep learning is important, but there is currently a lack of consensus on the best methods. Commonly used measures like peak signal-to-noise ratio (PSNR) and structural similarity index (SSIM) reflect perceptual similarity but not quantitative accuracy. Some studies have evaluated quantitative accuracy by comparing radioactivity concentration, SUV, contrast recovery, etc. However, more work is needed to establish standardized evaluation methods. A limitation is the lack of large PET image datasets to train deep learning models. Creating shared public databases could enable more transfer learning. However, given how emergent this post-COVID neurological dysfunction phenomenon is, such repositories are simply not available yet. As with many cutting edge applications of AI to new medical contexts, progress often starts from limited datasets. Efforts like the UK Biobank imaging dataset [33,34,35] on post-COVID neurological deficits demonstrate feasibility. With more patients being scanned, open data sharing and global coordination amongst researchers are absolutely key—and achievable. For optimal AI development, consolidated image repositories must be the crucial first step. Alternative techniques like unsupervised, self-supervised, and weakly supervised learning may help with limited data. Transformers have potential for breakthroughs in PET image generation, as they have in natural language processing. Attention-based models like BERT and XLNet could be applied to PET images. Multimodal PET/MR data alignment can introduce errors. Systematic evaluation of the effect of PET-MR alignment errors on deep learning performance is needed.

In summary, key challenges are developing standardized quantitative evaluation methods, creating large public PET image datasets, and exploring alternative deep learning techniques that require less data. Evaluating the impacts of multimodal data alignment is also important in future work.

## 9. Conclusions

Cutting-edge PET/FDG neuroimaging combined with AI analysis offers tremendous potential to elucidate the neurological impacts of Long COVID. AI techniques including CNNs and GANs can detect subtle patterns in PET data that provide insights into brain inflammation, hypometabolism, network dysfunction, and cognitive decline associated with Long COVID. Although still an emerging application, the integration of AI and advanced imaging could transform our understanding of Long COVID’s effects on the brain, enabling better diagnosis, prognostics, treatments, and eventually prevention. However, rigorous validation and attention to responsible and ethical AI development remain imperative as these technologies progress from bench to bedside. By harnessing the synergy between AI and neuroimaging, researchers seek to unravel the neurological complexities of Long COVID and meaningfully improve patient outcomes.

## Data Availability

Not applicable.
